# Novel reciprocal interaction of lncRNA HOTAIR and miR‐214‐3p contribute to the solamargine‐inhibited PDPK1 gene expression in human lung cancer

**DOI:** 10.1111/jcmm.14649

**Published:** 2019-09-01

**Authors:** Qing Tang, Fang Zheng, Zheng Liu, JingJing Wu, XiaoSu Chai, CuenXa He, Liuning Li, Swei Sunny Hann

**Affiliations:** ^1^ Laboratory of Tumor Biology The Second Clinical Collage of Guangzhou University of Chinese Medicine Guangzhou China; ^2^ Department of Medical Oncology The Second Clinical Collage of Guangzhou University of Chinese Medicine Guangzhou China; ^3^ Guangdong Provincial Key Laboratory of Clinical Research on Traditional Chinese Medicine Syndrome The Second Clinical Collage of Guangzhou University of Chinese Medicine Guangzhou China

**Keywords:** HOTAIR, human lung cancer cells, MiR‐214‐3p, PDPK1, solamargine

## Abstract

Solamargine (SM) has been shown to have anti‐cancer properties. However, the underlying mechanism involved remains undetermined. We showed that SM inhibited the growth of non‐small cell lung cancer (NSCLC) cells, which was enhanced in cells with silencing of long non‐coding RNA (lncRNA) HOX transcript antisense RNA (HOTAIR), while it overcame by overexpression of HOTAIR. In addition, SM increased the expression of miR‐214‐3p and inhibited 3‐phosphoinositide‐dependent protein kinase‐1 (PDPK1) gene expression, which was strengthened by miR‐214‐3p mimics. Intriguingly, HOTAIR could directly bind to miR‐214‐3p and sequestered miR‐214‐3p from the target gene PDPK1. Intriguingly, overexpression of PDPK1 overcame the effects of SM on miR‐214‐3p expressions and neutralized the SM‐inhibited cell growth. Similar results were observed in vivo. In summary, our results showed that SM‐inhibited NSCLC cell growth through the reciprocal interaction between HOTAIR and miR‐214‐3p, which ultimately suppressed PDPK1 gene expression. HOTAIR effectively acted as a competing endogenous RNA (ceRNA) to stimulate the expression of target gene PDPK1. These complex interactions and feedback mechanisms contribute to the overall effect of SM. This unveils a novel molecular mechanism underlying the anti‐cancer effect of SM in human lung cancer.

## 1 INTRODUCTION

Lung cancer is the number one cause of cancer‐related deaths worldwide.^1^ Non‐small cell lung cancer (NSCLC), which accounts for 85% of lung cancer cases, is the most common cause of cancer deaths. Over the last decades, significant improvement in the management of advanced stages of NSCLC was primarily due to an increased understanding of the molecular heterogeneity, drivers of lung cancer initiation and progression as well as therapeutics attributable to better treatment outcomes. However, despite these improvements, the 5‐year survival rate has shown little or no change.^1^, ^2^ Thus, there is a greater need to search for alternative and novel therapeutic modalities which will enhance the treatment efficacy in lung cancer patients.

Natural phytochemicals derived from medicinal plants have gained significant recognition in the control of carcinogenesis and are considered as a novel approach in the prevention and treatment of cancer. Solamargine (SM), a natural glycoalkaloid extracted from the fruits of *Solanum lycocarpum*, has been shown to be effective against several types of cancers.^3^, ^4^, ^5^, ^6^ It induced apoptosis via the mitochondrial pathway and altered the level of apoptosis‐associated proteins in human cholangiocarcinoma cells.^4^ SM caused the suppression of lung cancer cell growth in vitro and in vivo by increasing insulin‐like growth factor binding protein 1 (IGFBP1) expression through activation of the signal transducer and activator of transcription 3 (Stat3)/SP1/forkhead box O3a (FOXO3a) axis pathways.^7^ In addition, we have previously reported that SM can inhibit the growth of prostate cancer cells via AMP‐activated protein kinase (AMPK) α‐mediated inhibition of p65 and cell surface‐associated mucin 1 (MUC1) in vitro and in vivo.^8^ Regardless, the detailed mechanism underlying the anti‐lung cancer effects of SM remains to be elucidated.

Long non‐coding RNAs (lncRNAs) are key modulators of various pathological processes in human cancers. Among these, the oncogenic HOX transcript antisense intergenic RNA (HOTAIR), an approximately 2.2 kb lncRNA transcribed from the HOXC locus, is reported to be associated with growth and invasion of several types of cancer.^9^, ^10^, ^11^, ^12^ HOTAIR stimulated the migration and invasion of cervical cancer cells through miR‐206‐mediated regulation of transcription of the megakaryoblastic leukaemia 1 (MKL1).^10^ In addition, HOTAIR has been postulated to represent a biomarker of lung cancer because its upregulation positively correlates with metastasis, drug resistance and poor survival in lung cancer patients.^13^ Thus, HOTAIR is considered as a potential biomarker and therapeutic target in cancers.^13^, ^14^, ^15^ Nevertheless, the potential associations and the exact role of HOTAIR in lung cancer still remain undetermined.

Dysregulation of miRNA is associated with an increasing number of human diseases including cancer.^16^ Among these, miR‐214‐3p has been reported to be associated with growth, progression and survival in cancers.^17^, ^18^, ^19^, ^20^ miR‐214‐3p inhibited proliferation and cell cycle progression by targeting maternal embryonic leucine zipper kinase (MELK), also known as an oncogenic kinase and a key regulator in the malignancy and proliferation of cancer.^21^ Induced expression of miR‐214‐3p in oesophageal cancer cells resulted in a decrease in the expressions of survivin and CUG binding protein 1 (CUG‐BP1), an RNA‐binding protein, resulting in enhanced sensitivity of oesophageal cancer cells to cisplatin.^22^ The above results highlight the importance of miR‐214‐3p in cancer initiation and progression, suggesting that modulation of miR‐214‐3p may be a key therapeutic target for miRNA‐based cancer therapies.^23^ However, the role of miRNAs in the biology of lung cancer still remains unclear.

As a common upstream activator, 3‐phosphoinositide‐dependent protein kinase‐1 (PDPK1) activates multiple downstream effectors and promotes the development of various diseases including cancer.^24^, ^25^ Pharmacologic and genetic inhibition of PDPK1 resulted in the regression of tumour growth in vitro and attenuated the tumorigenesis in tumour models in vivo.^26^, ^27^, ^28^ Thus, PDPK1 may represent a novel and rational therapeutic candidate for preventing cancer.^29^, ^30^, ^31^ Of note, there are only limited numbers of studies demonstrating the link between miR‐214‐3p, HOTAIR and PDPK1. Therefore, a gap exists in our understanding of the role of this interaction in lung cancer.

Herein, we extended these studies and found that SM inhibited the growth of human lung cancer cells by suppressing PDPK1 expression through a reciprocal interaction between HOTAIR and miR‐214‐3p.

## 2 MATERIALS AND METHODS

### 2.1 Reagents and cell cultures

Antibodies specific for total PDPK1 were purchased from Cell Signaling Technology. miR‐214‐3p mimics and inhibitors were purchased from RiboBio Co. Ltd. miR‐214‐3p, U6 primers and HOTAIR siRNAs were provided by GenePharma. HOTAIR and GAPDH primers were obtained from Life Technologies. Lipofectamine 3000 reagent was supplied by Life Technologies. SM was purchased from Chengdu Must Bio‐technology Company, which was freshly diluted to the final concentration with culture medium before experiment. A549 and PC9 cells and BEAS‐2B cells were obtained from the Chinese Academy of Sciences Cell Bank of Type Culture Collection and authenticated for the absence of mycoplasma, genotypes and morphology by using a commercial kit provided by Guangzhou Cellcook Biotech Co. Ltd. Cells were cultured in RPMI 1640 (Life Technologies) supplemented with 10% foetal bovine serum at 37°C in a humidified atmosphere containing 5% CO2. A549‐Luc and A549‐PDPK1(+/+)‐Luc cells were provided by Guangzhou Land Technology Co and cultured in a medium containing geneticin Sulfate (Life Technologies). Cells at 70% confluence were trypsinized with 0.25% trypsin and used in all in vitro experiments.

### 2.2 Cell viability assay

The 3‐(4, 5‐dimethylthiazol‐2‐yl)‐2, 5‐diphenyltetrazolium bromide (MTT) assay was used to measure the cell viability as described previously.^32^ NSCLC cells (5 × 10^3^ cells/well) were counted and seeded into 96‐well microtiter plates, and treated with indicated doses of SM for up to 72 hours. After adding MTT solution (5 mg/mL), plates were incubated at 37°C for 4 hours followed by the addition of dimethyl sulfoxide. Absorbance was measured at 570 nm on a microplate reader (Perkin Elmer, Victor X5). Cell viability was calculated as the ratio of absorbance of sample/control. The cells treated with vehicle only (0.1% DMSO) served as a negative control and the control values were set to 1 by default.

### 2.3 EdU assay for cell proliferation

Non‐small cell lung cancer cell proliferation was assessed by Cell‐Light EdU Apollo 488 In Vitro Imaging Kit (RiboBio) according to instructions from the manufacturer. Briefly, after treatment with SM in 96‐well plates for 24 hours, the cells were incubated with EdU reagent for 2 hours and fixed in 4% paraformaldehyde for 30 minutes. Thereafter, cells were washed in glycine and incubated in 0.2% Trion X‐100 for 10 minutes followed by the addition of 1× Apollo reaction buffer. After washing in 0.5% Triton X‐100, the cells were stained with Hoechst (5 mg/mL). Images were taken under a BX53+DP72 Microscope (Olympus Corporation) and evaluated by Image‐Pro plus 6.0 image analysis software (Media Cybernetics, Inc). Per cent cell proliferation was calculated as: (EdU positive cells/Hoechst stained cells) × 100.

### 2.4 Flow cytometric analysis

Cell apoptosis was detected using Annexin V‐FITC/PI Apoptosis Detection Kit (BD Biosciences) according to the manufacturer's protocol. Briefly, the cells were treated with SM for 24 hours, washed with pre‐cooled phosphate‐buffered saline (PBS), and mildly trypsinized without the use of EDTA. Subsequently, the cells were harvested, resuspended in 500 μL of the binding buffer and incubated with 5 μL Annexin V‐FITC reagent for 15 minutes and 10 μL PI for 5 minutes at room temperature (RT) in the dark. Cell apoptosis was measured by flow cytometry (FC500, Beckman Coulter), and data were analysed by GraphPad Prism 5.0 software.

### 2.5 Cell invasion assay

The potential invasiveness of lung cancer cells was assessed using transwell plates with 10 mm diameter and 8 μm pore size polycarbonate membrane coated with a film of Matrigel (BD Biosciences) as reported previously.^33^ The upper compartment was seeded with 0.5 × 10^6^ cells and treated with or without SM. The lower compartment was filled with culture medium. After 24 hours incubation, non‐migrated cells were removed and migrated cells were fixed in 4% paraformaldehyde, and stained with crystal violet. Stained cells were counted and images were taken under Ts2RFL microscope (Nikon). The number of invaded cells is represented as percentage of control.

### 2.6 Quantitative real‐time RT‐PCR

First‐strand cDNA was synthesized from total RNA (2 μg) by reverse transcription using oligo‐dT primers and Superscript II reverse transcriptase (Invitrogen). A quantitative real‐time RT‐PCR (qRT‐PCR) assay was performed on an ABI 7500 Real‐Time PCR System (Applied Biosystems) for the quantification of HOTAIR and miR‐214‐3p transcripts. The sequences of the primers used were as follows: HOTAIR‐Forward: 5′‐GGTAGAAAAAGCAACCACGAAGC‐3′; Reverse: 5′‐ACATAAACCTCTGTCTGTGAGTGCC‐3′; miR‐214‐3p‐Forward: 5′‐CAATACTGACAGCAGGCACA‐3′ Reverse: 5′‐TATGGTTGTTCACGACTCCTTCAC‐3′ U6: Forward: 5′‐ATTGGAACGATACAGAGAAGATT‐3′ Reverse: 5′‐GGAACGCTTCACGAATTTG‐3′ GAPDH‐Forward: 5′‐AAGCCTGCCGGTGACTAAC‐3′; Reverse: 5′‐GCGCCCAATACGACCAAATC‐3′. The PCR conditions were as follows: 10 minutes at 95°C, followed by 40 cycles of 15 seconds at 95°C, and 1 minute at 60°C. Threshold values for each sample/primer pair are represented as mean ± standard error.

### 2.7 Determination of amplification efficiency

Reverse‐transcribed cDNA was diluted by 5 10‐fold serial dilutions. qRT‐PCR was performed to detect the expression of HOTAIR and miR‐214‐3p with the corresponding primers, and each sample was processed three times. A standard curve was generated by plotting the log values of dilution multiples on the *X*‐axis and the corresponding CT values on the *Y*‐axis. The amplification efficiency of the primers, *E*, was calculated as: *E* = 10^−1/slope^−1. The amplification efficiency in the range from 90% to 110% is generally considered to be adequate. Our results showed that amplification efficiency for HOTAIR and miR‐214‐3p was 100.99% and 100.34%, respectively.

### 2.8 Western blot analysis

Equal amounts of protein from whole cell lysates were dissolved in SDS‐sample buffers and separated on 10% SDS polyacrylamide gels. Membranes (Millipore) were incubated with antibodies against PDPK1 for 2 hours, washed and incubated with secondary rabbit IgG antibody conjugated to horseradish peroxidase (Cell Signaling Technology). The membranes were washed again and transferred to a freshly prepared enhanced chemiluminescence solution (Immobilon Western; Millipore). Protein bands were observed using the Gel Imagine System (Bio‐Rad). ImageJ software (National Institutes of Health) was used to quantify and compare the intensity of single band between the control and proteins of interest.

### 2.9 Transient transfection assays

Cells were seeded in 6‐well or 96‐well culture plates and grown to 60% confluency before transfection with the control or HOTAIR (pcDNA3.1‐HOTAIR) and PDPK1 plasmids (pCMV6‐PDPK1) using Lipofectamine 3000 reagent. miR‐214‐3p mimics or inhibitors were transfected with RiboFect CP reagent (RiboBio Co.) according to the manufacturer's instructions. Briefly, Lipofectamine 3000 was incubated with Opti‐MEM medium (Invitrogen) for 10 minutes followed by respective siRNA (up to 25 nmol/L) for an additional 15 minutes before adding to the cells. RiboFect CP regent was incubated the miRNA mimics or inhibitors for 15 minutes. After culturing the cells for up to 48 hours, the cells were washed and resuspended in fresh media in the presence or absence of SM for an additional 24 hours.

### 2.10 Luciferase reporter assay

The binding sites of HOTAIR and PDPK1 for miR‐214‐3p were obtained using bioinformatics prediction databases (TargetScan, http://www.targetscan.org/vert_72/, MiRBase, http://www.mirbase.org/, and MiRWalk http://mirwalk.umm.uni-heidelberg.de/). 3′‐UTR cDNA fragment of PDPK1 containing the wild‐type and mutated miR‐214‐3p binding sites, named pEZX‐MT05‐Luc‐PDPK1‐WT or pEZX‐MT05‐Luc‐PDPK1‐Mut and 3′‐UTR cDNA fragments of HOTAIR containing the wild‐type and mutated miR‐214‐3p binding sites, named pEZX‐MT05‐Luc‐HOTAIR‐WT or pEZX‐MT05‐Luc‐HOTAIR‐Mut were ordered from GeneCopoeia, Inc. The cells were transfected with the plasmids (1.25 μg/mL each) for 24 hours using Lipofectamine 3000 reagent and then treated with miR‐214‐3p mimics for an additional 48 hours. The preparation of cell extracts and measurement of luciferase activities were done by using Secrete‐Pair Dual Luminescence Assay Kit (GeneCopoeia, Inc) and were normalized with SEAP activity within each sample.

### 2.11 Cell immunofluorescence

Cells were fixed in paraformaldehyde and permeabilized with 0.1% Triton X‐100 (Sigma‐Aldrich) followed by incubation in normal goat serum for 30 minutes, and incubation with anti‐human PDPK1 antibody (1:100) overnight. After washing, the cells were incubated with secondary anti‐Rabbit IgG (H+L) antibody conjugated to Alexa Fluor 594 (A21442, Invitrogen, ThermoFisher) followed by washing and counterstaining with DAPI. Slides were mounted with FluorSave (Calbiochem). Fluorescence was detected under an Axio Observer Z1 immunofluorescence microscope (Carl Zeiss Inc).

### 2.12 RNA immunoprecipitation (RIP) assay

RNA immunoprecipitation (RIP) assay was performed using the Magna RIP RNA‐Binding Protein Immunoprecipitation Kit (Millipore) following instruction from the manufacturer. Briefly, after washing in PBS, the cells were lysed in RNA lysis buffer containing RNase inhibitor. The cells lysates were incubated with magnetic beads coated with the specific PDPK1 antibody (Abcam), anti‐Ago2 antibody (Millipore) or the negative control IgG (Millipore). Finally, the expression of HOTAIR, miR‐214‐3p, PDPK1 and Ago2 was measured by qRT‐PCR as well as Western blot.

### 2.13 Xenograft tumours and bioluminescent imaging

Animal studies were performed according to the protocols approved by the Institutional Animal Care and Use Committee of Guangdong Provincial Hospital of Chinese Medicine (Ethics Approval Number 2017037) and the National Institutes of Health guidelines for the care and use of Laboratory animals (NIH Publications No. 8023, revised 1978). Nude mice (aged 4‐6 weeks) purchased from Beijing Vital River Experimental Animal Co. Ltd. were maintained at the Animal Center of Guangdong Provincial Hospital of Chinese Medicine. A549‐Luc or A549PDPK1+/+‐Luc (2 × 10^6^) were injected subcutaneously into the nude mice. Xenografts were allowed to grow when the initial measurements were made with calipers. For bioluminescence imaging (BLI), mice were anesthetized by inhalation of 2% isoflurane. The substrate D‐luciferin (Caliper Life Sciences) was injected into the peritoneal cavity of the mice at a dose of 150 mg/kg. The intensity of the BLI signal was determined using the IVIS‐200 Imaging System (Xenogen/Caliper). Mice were then randomly divided into the control and SM groups (n = 10/group), and SM was injected intraperitoneally daily at a dose of 8 mg/kg for up to 25 days based on our previous reports and others,^7^, ^34^, ^35^ which showed significant inhibitory effect of SM on tumour growth without apparent toxicity. Tumour volume was calculated using the formula for a spheroid: volume = (width^2^ × length) × 0.5. Bioluminescence is expressed as photons/s. Body weights of mice were measured once a week. All mice were sacrificed on the 25th day in accordance with the Guidelines for the Care and Use of Laboratory Animals. At the end of the experiments, xenograft tumours were isolated, and expression of HOTAIR, miR‐214‐3p and PDPK1 was determined by qRT‐PCR as well as Western blot.

### 2.14 Statistical analysis

All in vitro experiments were performed at least three times. Statistical analysis was carried out using GraphPad Prism version 5.04 for Windows (GraphPad Software). Pair wise comparisons were done by paired two‐tailed *t* test, Mann‐Whitney test or Fisher exact test. The data in most graphs are presented relative to the control. *P* values <.05 were considered significant.

## 3 RESULTS

### 3.1 SM‐inhibited proliferation of NSCLC cells via inhibition of HOTAIR

**Figure 1:**
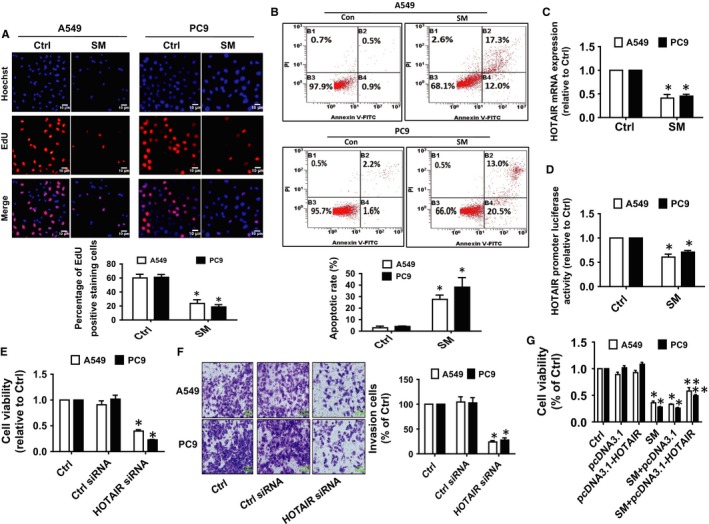
SM‐inhibited proliferation of NSCLC cells via inhibition of HOTAIR. A, A549 and PC9 cells were treated with SM (6 μmol/L) for 48 h, followed by determination of cell growth with the Cell‐Light EdU DNA cell proliferation kit. The image was magnified 10×. Hoechst was used to stain all the nuclei. At least five captured fields were randomly selected, and the percentage of EdU positive cells = (EdU positive cells/Hoechst stain cells) × 100. Scale bars, 10 μm. B, A549 and PC9 cells were treated with SM (6 μmol/L) for 24 h, and then, cells were harvested for Flow cytometric analysis by using the Annexin V‐FITC/PI Apoptosis Detection Kit. The B1 quadrant showed for percentage of dead cells, B3 quadrant represented percentage of normal cells, B2 and B4 quadrant indicated the percentage of late and early apoptosis, respectively. C, A549 and PC9 cells were treated with SM (6 μmol/L) for 24 h, and the expression levels of HOTAIR were measured via qRT‐PCR. D, A549 and PC9 cells were transfected with the control or the HOTAIR promoter vectors for 24 h followed by measuring luciferase activity using Secrete‐Pair™ Dual Luminescence Assay Kit as described in the Materials and Methods section. E, F, A549 and PC9 cells were transfected with the control or HOTAIR siRNAs (25 nmol/L) for up to 48 h followed by determining the cell growth and invasion as determined by MTT and in vitro invasion assays. Scale bars, 10 μm. G, A549 and PC9 cells were transfected with the control or the HOTAIR expression vectors (1.25 μg/mL each) for up to 48 h, followed by determining the cell growth via MTT assays. Values and bar graphs are presented as the mean ± SD of three independent experiments performed. *Indicates significant difference from the control group (*P* < .05). **Indicates significant difference from the SM alone (*P* < .05)

Previous reports showed that SM significantly inhibited the growth of NSCLC cells via several mechanisms.^7^, ^34^ In the current study, we demonstrated that percentage of EdU positive NSCLC cells was significantly reduced in the SM‐treated group compared with the control group (Figure 1A). This further confirmed the inhibitory effect of SM on the growth of NSCLC cells. Moreover, SM induced a high magnitude of apoptosis, as determined by staining with Annexin V/PI and flow cytometry analysis (Figure 1B).

Studies have demonstrated the important roles of lncRNAs such as HOTAIR in growth and progression of cancers.^13^, ^36^ Herein, the results unveiled that SM significantly inhibited the expression as well as the promoter activity of HOTAIR (Figure 1C,D). Moreover, siRNA mediated silencing HOTAIR significantly inhibited the growth and invasion of A549 and PC9 cells as determined by MTT and in vitro invasion assays (Figure 1E,F), respectively. Overexpression of HOTAIR, on the other hand, diminished the inhibitory effect of SM on cell growth (Figure 1G). Taken together, our results demonstrated that HOTAIR may be an important target of SM and that inhibition of HOTAIR is involved in the SM‐mediated inhibition of lung cancer cells.

### 3.2 SM increased miR‐214‐3p expression in NSCLC cells

**Figure 2:**
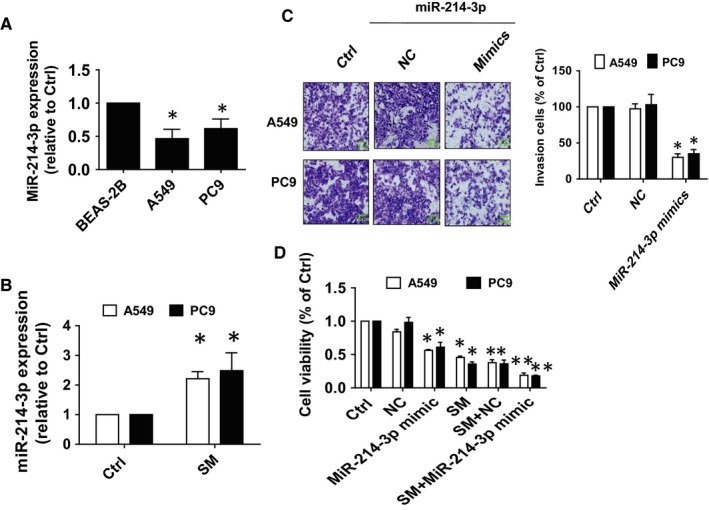
SM increased miR‐214‐3p expression in NSCLC cells. A, Total RNA was isolated from A549, PC9 and normal human bronchial epithelial cells (BEAS‐2B) and processed for determining the mRNA levels of miR‐214‐3p via qRT‐PCR. B, A549 and PC9 cells were treated with SM (6 μmol/L) for up to 24 h, followed by measuring miR‐214‐3p via qRT‐PCR. C, D, A549 and PC9 cells were treated with the control or the miR‐214‐3p mimics (100 nmol/L) for up to 48 h followed by determining cell growth and invasion via MTT and in vitro invasion assays. Scale bars, 10 μm. Values and bar graphs are presented as the mean ± SD of three independent experiments performed. *Indicates significant difference from the control group (*P* < .05). **Indicates significant difference from the SM alone (*P* < .05)

To further dissect the mechanism of the inhibitory effect of SM on lung cancer cells, we searched for potential miRNAs that may link HOTAIR and other target genes. We observed reduced transcript abundance of miR‐214‐3p in A549 and PC9 cells compared with normal human bronchial epithelial cell (BEAS‐2B; Figure 2A), however, in the presence of SM there was a significant increase in expression of miR‐214‐3p (Figure 2B). Furthermore, miR‐214‐3p mimics significantly inhibited the growth and invasiveness of A549 and PC9 cells as compared with the control group (Figure 2C,D). These results confirmed that miR‐214‐3p functions as a tumour suppressor role and suggested that miR‐214‐3p may enhance the SM‐mediated inhibition of NSCLC cell growth.

### 3.3 Interaction between HOTAIR and miR‐214‐3p was involved in SM‐inhibited cell growth

**Figure 3:**
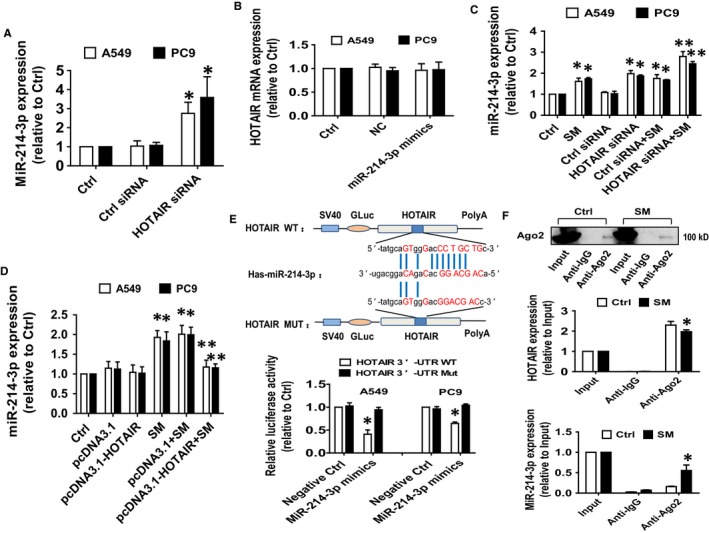
The interaction between HOTAIR and miR‐214‐3p was involved in the SM‐inhibited cell growth. A, A549 and PC9 cells were transfected with the control or HOTAIR siRNAs (25 nmol/L) for up to 48 h followed by determining the expression of miR‐214‐3p via qRT‐PCR. B, A549 and PC9 cells were treated with the control or the miR‐214‐3p mimics (100 nmol/L) for up to 48 h followed by determining HOTAIR expression via qRT‐PCR. C, A549 and PC9 cells were transfected with the control or HOTAIR siRNAs for up to 24 h followed by exposure the cells to SM for an additional 24 h, and afterwards, miR‐214‐3p expressions were detected via qRT‐PCR. D, A549 and PC9 cells were transfected with the control or the HOTAIR expression vectors (1.25 μg/mL each) for up to 24 h followed by exposure the cells to SM for an additional 24 h, and afterwards, miR‐214‐3p expressions were detected via qRT‐PCR. E, The luciferase reporter constructs containing a wild‐type and mutant HOTAIR sequences were shown (upper panel). A549 and PC9 cells were transfected with the HOTAIR 3′‐UTR‐WT or HOTAIR 3′‐UTR‐Mut vectors (1.25 μg/mL each) for 24 h, and then treated with the miR‐214‐3p mimics (100 nmol/L) or miR‐negative control (NC) for an additional 48 h. Afterwards, the luciferase activity was detected using Secrete‐Pair™ Dual Luminescence Assay Kit as described in the Materials and Methods section (lower panel). F, Cell lysates from A549 cells were incubated with Ago2 antibody‐coated magnetic beads. Precipitates ware subjected to Western blot for Ago2 protein and qRT‐PCR for detecting HOTAIR and miR‐214‐3p expression levels. Preimmune IgG and input from cell extracts were used as controls. The figures are representative cropped gels/blots that have been run under the same experimental conditions. Values and bar graphs are presented as the mean ± SD of three independent experiments performed. *Indicates significant difference from the control group (*P* < .05). **Indicates significant difference from the SM alone (*P* < .05)

Next, we began to test the biological significance of the interaction between HOTAIR and miR‐214‐3p in mediating the effect of SM. Results showed that the expression of miR‐214‐3p was significantly higher in cells with silenced HOTAIR compared with the control siRNA‐transfected cells (Figure 3A). Conversely, mimics of miR‐214‐3p had no effect on HOTAIR expression (Figure 3B). Moreover, we found that silencing HOTAIR further strengthened SM‐induced miR‐214‐3p expression (Figure 3C), while overexpression of HOTAIR reversed SM‐induced miR‐214‐3p expression (Figure 3D).

By utilizing bioinformatics prediction databases, we found that miR‐214‐3p has a classical and conservative binding site in the 3′‐UTR region of HOTAIR (Figure 3E). We generated a 3′‐UTR cDNA fragment of HOTAIR containing the wild‐type and mutated miR‐214‐3p binding sites (Figure 3E). We found that the interaction of miR‐214‐3p with the wild‐type 3′‐UTR cDNA fragment of HOTAIR significantly decreased the luciferase activities of HOTAIR in A549 and PC9 cells compared with the negative control group (Figure 3E) suggesting a direct inhibitory effect of miR‐214‐3p on HOTAIR expression. Furthermore, RIP assay showed that Ago2 protein was successful pulled down by Ago2 antibody‐coated magnetic beads (Figure 3F). And qRT‐PCR analysis revealed that both HOTAIR and miR‐214‐3p were enriched in the Ago2‐containing beads and were higher compared with the input group (Figure 3F). Moreover, the physical binding of HOTAIR to miR‐214‐3p was also significantly affected in the presence of SM (Figure 3F). These results suggested that the physical interaction between HOTAIR and miR‐214‐3p may also play an additional role in mediating the anti‐carcinogenic effect of SM.

### 3.4 SM‐inhibited PDPK1 expression via correlation and interaction of HOTAIR and miR‐214‐3p

**Figure 4:**
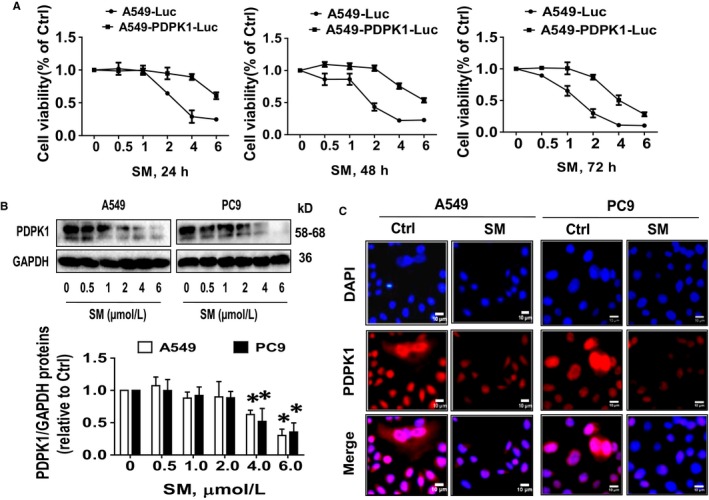
SM‐inhibited PDPK1 expression and reduced cell growth through reduction of PDPK1. A, A549‐luc and A549‐PDPK1‐luc cells (4 × 10^3^ cells/well）were seeded into the 96‐well microplate and treated with increased concentration of SM for up to 72 h followed by measuring the cell growth (viability) at different time zone via MTT assays as described in the Material and Method section. B, C, A549 and PC9 cells were treated with indicated doses of SM for 24 h followed by detecting PDPK1 protein expression via Western blot and Cell immunofluorescence assays as described in the Materials and Methods section. Scale bars, 10 μmol/L. The figures are the representative cropped gels/blots that have been run under the same experimental conditions. Values and bar graphs are presented as the mean ± SD of three independent experiments performed. *Indicates significant difference from the control group (*P* < .05)

Previously, we demonstrated an important role of PDPK1 in cancer growth with the implication that targeting PDPK1 may be a potential treatment of lung cancer.^33^, ^37^ In line with this, overexpression of PDPK1 in A549PDPK1+/+‐Luc cells not only showed more growth regression, it also significantly resisted the SM‐inhibited growth as compared with the wild‐type A549‐Luc cells (Figure 4A). Both Western blot and immunofluorescence confirmed that SM reduced PDPK1 protein expression (Figure 4B,C).

**Figure 5:**
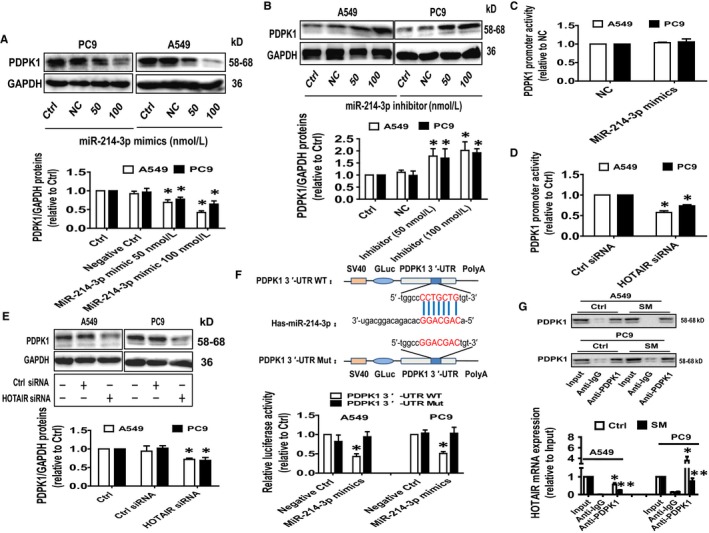
The regulation and interaction of HOTAIR and miR‐214‐3p contributed to the inhibition of PDPK1 expression treated with SM. A, B, A549 and PC9 cells were treated with the negative control, miR‐214‐3p mimics (50‐100 nmol/L) or miR‐214‐3p inhibitors (50‐100 nmol/L) for 24 h followed by measuring the PDPK1 protein expression via Western blot. C, D, A549 and PC9 cells were treated with the control or the miR‐214‐3p mimics (100 nmol/L), or the control and HOTAIR siRNAs, and the PDK1 promoter construct ligated to luciferase reporter gene and an internal control secreted alkaline phosphatase for up to 48 h. Afterwards, the PDPK1 promoter activity was detected using Secrete‐Pair™ Dual Luminescence Assay Kit as described in the Materials and Methods section. E, A549 and PC9 cells were treated with the control or HOTAIR siRNAs, and the PDK1 protein expression was determined via Western blot. F, The luciferase reporter constructs containing the wild‐type and mutant binding sites in 3′‐UTR region of PDPK1 mRNA were shown (upper panel). A549 and PC9 cells were transfected with the PDPK1 3′UTR‐WT or PDPK1 3′‐UTR‐Mut vectors (1.25 μg/mL each) for 24 h and then treated with the miR‐214‐3p mimics (100 nmol/L) or miR‐negative control (NC) for an additional 48 h. Afterwards, the luciferase activity was detected using Secrete‐Pair™ Dual Luminescence Assay Kit as described in the Materials and Methods section (lower panel). G, Cell lysates from both A549 and PC9 cells treated with SM (6 μmol/L) for 24 were immunoprecipitated using the anti‐PDPK1 monoclonal antibody and IgG1 isotype control. The relative enrichment of HOTAIR was determined after normalized to the input via qRT‐PCR. The figures are the representative cropped gels/blots that have been run under the same experimental conditions. Values in bar graphs were given as the mean ± SD from three independent experiments. *Indicates significant difference as compared to the untreated control group (*P* < .05). **Indicates significant difference from the SM alone (*P* < .05)

In order to validate our findings and further identify the relevant targets of HOTAIR and miR‐214‐3p association, we treated the NSCLC cells with miR‐214‐3p mimics and determined PDPK1 protein expression. Results showed that miR‐214‐3p mimics reduced whereas miR‐214‐3p inhibitors increased PDPK1 protein expression (Figure 5A,B). While over‐expressing miR‐214‐3p had no effect, silencing HOTAIR resulted in decreased PDPK1 promoter activity (Figure 5C,D). Note that the latter also suppressed PDPK1 protein expression (Figure 5E). Together, these results suggested that while miR‐214‐3p may regulate PDPK1 post‐transcriptionally, HOTAIR may regulate both transcriptionally and translationally.

To further delineate the functional role of miR‐214‐3p and whether PDPK1 is its downstream target, we searched bioinformatics prediction databases and found a classical and conservative binding site for miR‐214‐3p in 3′‐UTR region of PDPK1 (Figure 5F). We observed that the binding of miR‐214‐3p mimics to the wild‐type 3′‐UTR cDNA fragment of PDPK1 significantly reduced the luciferase activity in comparison with the mutated 3′UTR or negative control/ scrambled mimics (Figure 5F). The results suggested that PDPK1 is a downstream target of miR‐214‐3p. Finally, RIP assay demonstrated a physical interaction between HOTAIR and PDPK1 protein which was inhibited by SM (Figure 5G). These results suggested that SM exerts its effect on the interaction between HOTAIR and PDPK1 protein by suppressing PDPK1 expression.

### 3.5 Overexpression of PDPK1 neutralized the effect of SM on HOTAIR and miR‐214‐3p expressions, and cell growth

**Figure 6:**
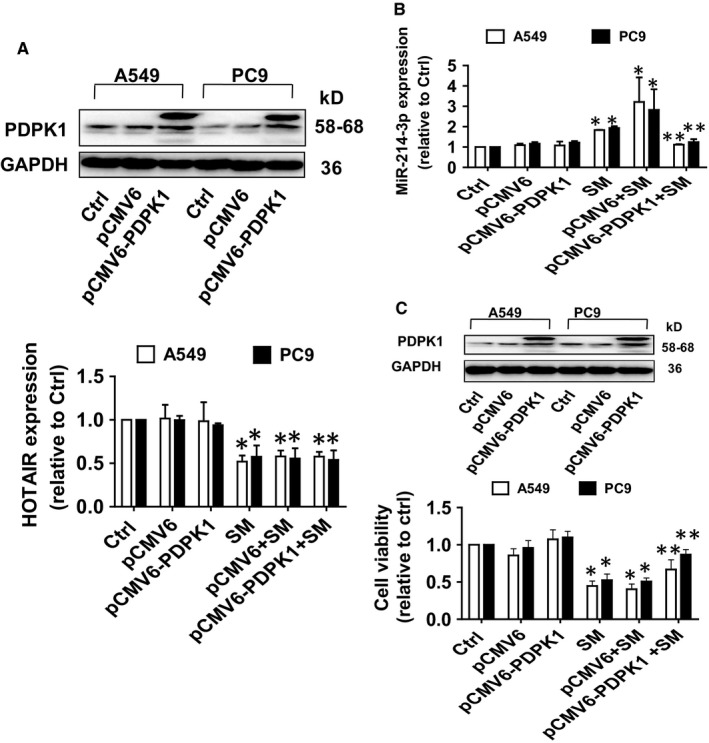
Overexpression of PDPK1 neutralized the effect of SM on HOTAIR and miR‐214‐3p expressions, and cell growth. A‐C, A549 and PC9 cells were transfected with the control or overexpressed PDPK1 vector for 24 h before exposure of the cells to SM (6 μmol/L) for an additional 24 h. Afterwards, the expression of HOTAIR, miR‐214‐3p, and cell growth was measured via qRT‐PCR and MTT assays as described in the Materials and Methods section, respectively. Values in bar graphs were given as the mean ± SD from three independent experiments. *Indicates significant difference as compared to the untreated control group (*P* < .05). **Indicates significant difference from the SM alone (*P* < .05)

In order to confirm the role PDPK1 in the interaction of HOTAIR and miR‐214‐3p, we showed that overexpression of PDPK1 neutralized the effects of SM on miR‐214‐3p, but not HOTAIR, expression (Figure 6A,B). In addition, overexpressed PDPK1 overcame the SM‐inhibited growth in NSCLC cells (Figure 6C). Together, the findings indicate the presence of a feedback loop between PDPK1 and miR‐214‐3p, and the interactive regulatory axis among HOTAIR, miR214‐3p and PDPK1 contributing to the inhibitory activity of SM on growth of NSCLC cells.

### 3.6 SM‐inhibited tumour growth and regulated the expressions of HOTAIR, miR‐214‐3p and PDPK1 in vivo

**Figure 7:**
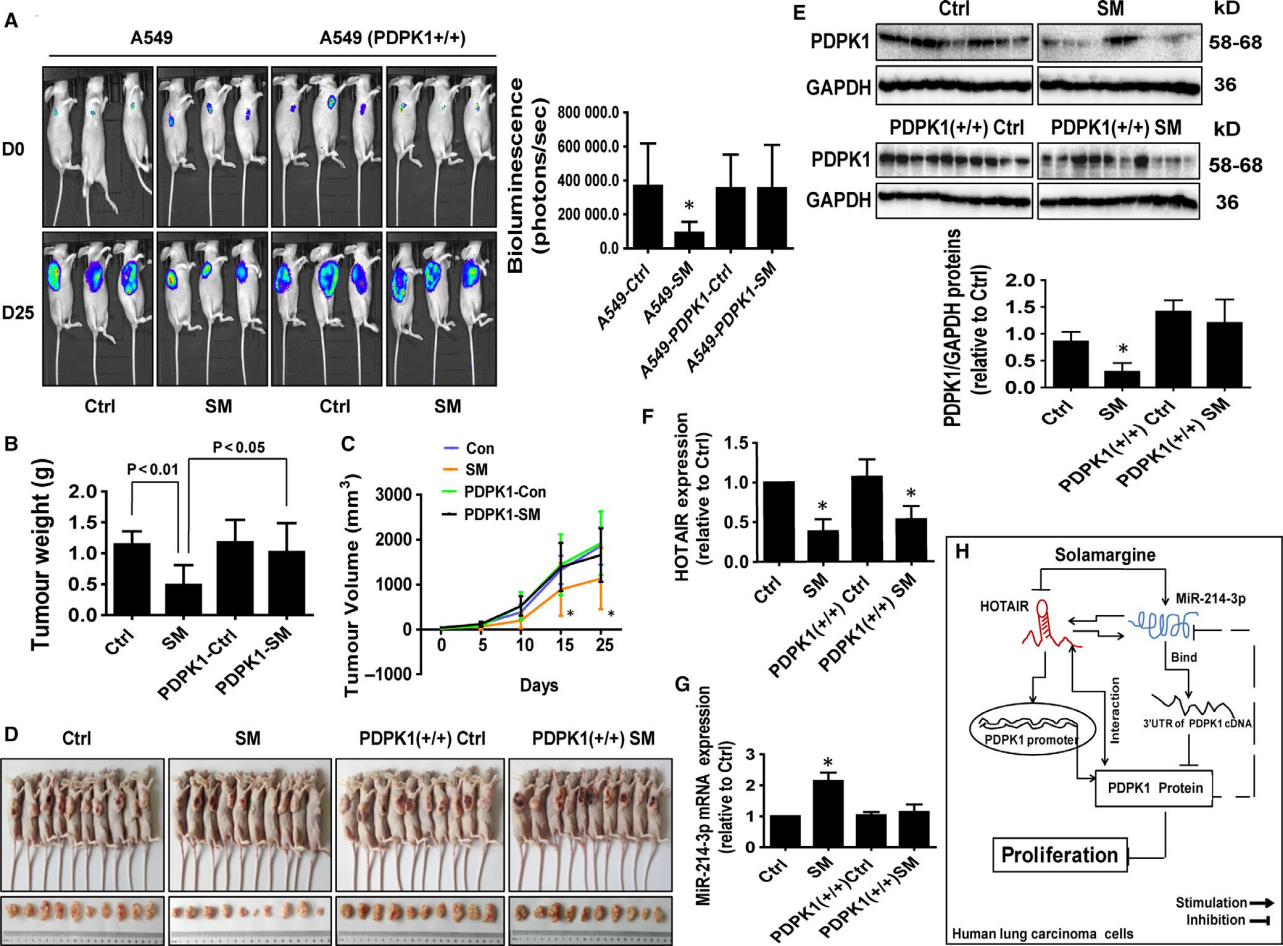
SM‐inhibited tumour growth and regulated expressions of HOTAIR, miR‐214‐3p and PDPK1 in vivo. A, The tumour growth was monitored by injecting luciferin followed by measuring bioluminescence signals. Representative images are shown. B, C, The xenografts were harvested on day 25, and the weight (B) and volume (C) of tumours in wild‐type A549 cells (A549‐Luc) and PDPK1 stable transfected cells (A549PDPK1+/+‐Luc) were measured. D, The photographs of the SM (8 mg/kg) and the vehicle‐treated xenografts derived from nude mice in A549‐Luc and PDPK1 stable transfected cells (A549PDPK1+/+‐Luc) were shown. E‐G, At the end of the experiments, the xenograft tumours were isolated and processed for detecting HOTAIR, miR‐214‐3p and PDPK1 via qRT‐PCR and Western blot, respectively. The figures are the representative cropped gels/blots that have been run under the same experimental conditions. Values in bar graphs were given as the mean ± SD from three independent experiments. *indicates significant difference as compared to the untreated control group (*P* < .05). H, The diagram shows that SM inhibits NSCLC cell growth through reciprocal interaction between HOTAIR and miR‐214‐3p, this result in inhibition of PDPK1 expression. This complex interaction and feedback regulatory axis contribute to the overall effect of SM in vitro and in vivo

We used A549‐Luc and A549‐PDPK1 (+/+)‐Luc cells to examine whether overexpression of PDPK1 resisted the effect of SM on tumour growth in vivo. We found that SM significantly inhibited tumour growth (Figure 7A). However, the inhibitory effect was reduced with A549‐PDPK1 (+/+)‐Luc cells (Figure 7A). In addition, SM caused significant decrease in tumour weight and sizes in the established A549 xenograft tumours compared with that of the vehicle‐treated control animals (Figure 7B‐D). Note that there was less effect with A549‐PDPK1 (+/+)‐Luc cells (Figure 7B‐D). Moreover, consistent with the in vitro data, SM‐induced miR‐214‐3p expression, while reduced the expressions of HOTAIR and PDPK1 in fresh tumours harvested from the aforementioned experiments as compared with the control group (Figure 7E‐G). As seen before, xenograft tumours with A549‐PDPK1 (+/+)‐Luc cells showed less inhibitory effects (Figure 7E‐G). Taken together, these results indicated that both in vitro and in vivo studies show similar effects of SM on lung tumour growth and expressions of relevant molecules.

## 4 DISCUSSION

Solamargine, a natural photochemical component extracted from the fruits of *Solanum lycocarpum*, has been shown to have anti‐cancer properties.^4^, ^5^, ^38^ Studies from our and other laboratories have demonstrated that SM inhibits the growth of cancer cells including lung by regulating several molecules and signalling pathways.^4^, ^34^, ^35^, ^36^, ^38^ We recently found that atractylenolide 1, one of major bioactive compounds of Atractylodis macrocephalae, inhibited growth of lung cancer cells through ERK1/2‐mediated suppression of Stat3 and SP1, resulting in inhibition of PDPK1 gene expression.^37^ It is well known that RNA species, such as lncRNAs and miRNAs, are extensively studied because of their function as gene regulators in both normal biological and pathological processes, such as tumorigenesis.^39^ In the current study, we provided new evidence on a reciprocal inter‐regulation between the lncRNA HOTAIR and miR‐214‐3p. These interactions contribute to the inhibition of PDPK1 expression, and, together with a feedback regulatory axis, synergistically enhance the overall anti‐lung cancer effect of SM.

We demonstrated a role of HOTAIR in the SM‐mediated inhibition of lung cancer cell growth. Aberrant increase in HOTAIR expression is positively correlated with growth, progression, drug resistance, recurrence and poor prognosis; all of which are mediated by regulation of several downstream targets through multiple signalling pathways.^40^, ^41^, ^42^ Our results confirmed that HOTAIR is an important target gene of SM in lung cancer cells and that inhibition of HOTAIR by SM is involved in the inhibition of NSCLC cell growth. In line with this, HOTAIR was highly expressed in NSCLC cells and involved in cell migration, growth, invasion and metastasis.^13^, ^43^, ^44^, ^45^ The aberrant HOTAIR expression is expected to be considered as a potential biomarker for patients with NSCLC.^46^ We also observed the critical role of miR‐214‐3p as a tumour suppressor in mediating the effect of SM on NSCLC cell growth. MiR‐214‐3p has been reported to be involved in several biological functions and associated with growth, apoptosis, progression and survival.^17^, ^18^, ^19^ This shows that miR‐214‐3p may be a key therapeutic target for miRNA‐based therapies for cancer.^23^ In addition, HOTAIR also affects the miRNAs‐mediated suppression of target gene expression by competitive binding to miRNAs. Our findings suggested that not only the regulation but also the reciprocal and physical interaction between HOTAIR and miR‐214‐3p in the presence of SM might be involved in the anti‐lung cancer effect of SM. A limited number of studies have shown the interaction of HOTAIR with miRNAs in influencing cancer cell growth,^47^, ^48^ and one study reported the link between HOTAIR and miR‐214‐3p.^49^ This unravelled a novel mechanism underlying the anti‐cancer effect of SM. HOTAIR may also act as a ceRNA for miRNAs thereby modulating the endogenous target gene expression and subsequent pathways in cancer cell growth.^48^, ^50^ Future studies are required to test and confirm the role of HOTAIR in primary cells of NSCLC. Of note, the physical binding of HOTAIR to miR‐214‐3p affected in the presence of SM was not strong, in particular in A549 cells, although the statistical difference was observed. We believe that the true significance of this association and the details of the underlying mechanism need to be determined in the future.

PDPK1, a master regulatory protein kinase and a member of the AGC protein kinase family, activates multiple downstream effectors implicated in various diseases including cancer.^24^ Inhibition of PDPK1 reduced proliferation and progression and attenuated tumorigenesis in vivo in several tumour models.^26^, ^27^, ^28^ Our results showed that, while A549 cells with stably expressing PDPK1 showed more aggressive growth, overexpression of PDPK1 significantly countered the inhibitory effects of SM indicating and confirming the tumorogenic properties of this molecule. Furthermore, our result implied that PDPK1 was regulated post‐transcriptionally by miR‐214‐3p and both transcriptionally and translationally by HOTAIR. As miRNAs regulate their target genes by binding to the 3′‐UTR resulting in either mRNA degradation or inhibition of translation,^51^, ^52^ we established that miR‐214‐3p directly inhibited PDPK1 gene expression by binding to specific sequences in 3′‐UTR. Limited studies have demonstrated the association and interaction between miR‐214‐3p, HOTAIR and PDPK1. Our findings indicated that both miR‐214‐3p and HOTAIR act as upstream regulators of PDPK1 gene expression in the presence of SM in lung cancer cells. In addition, SM reduced HOTAIR binding to PDPK1 protein implying that this interaction could lead to a reduction of genes, including PDPK1 that may be involved in the effect of SM in anti‐lung cancer effects. Together, our results suggested that HOTAIR may function as a competing endogenous RNA and sequester miR‐214‐3p from its target gene, PDPK1. These novel interactions and correlations unveiled a previously unknown mechanism underlying the anti‐carcinogenic effect of SM in lung cancer.

The alignment of the in vivo results with the in vitro observations further confirms that the inhibitory effects of SM on lung tumour growth are mediated through the interaction and feedback regulatory axis of miR‐214‐3p, HOTAIR and PDPK1. The doses of SM used were based on our previous reports^8^, ^34^ and another study,^35^ which showed significant inhibitory effects on tumour growth without noticeable toxicity. Our findings suggested that SM‐inhibited human lung cancer cell growth via inhibition of the PDPK1 and HOTAIR, and induction of miR‐214‐3p signalling axis.

In summary, our results show that SM inhibits NSCLC cell growth through reciprocal interaction between HOTAIR and miR‐214‐3p. This complex interaction of HOTAIR and miR‐214‐3p, and the regulatory feedback axis contribute to the overall anti‐lung cancer effects of SM in vitro and in vivo (Figure 7H). These findings improve our understanding of the underlying mechanisms involved in the anti‐cancer effect of SM and provide novel molecular targets for the treatment of human lung cancer.

## CONFLICT OF INTEREST

The authors declare that they have no competing interests.

## AUTHOR CONTRIBUTIONS

SSH conceived of the study, participated in its design and coordination, draft and finalized the manuscript. QT carried out the cell growth, siRNA, Western blot assays, transfection, luciferase reporter assays, RIP and statistical analysis. FZ, ZL and JJW participated in performing the cell viability, siRNA, transfection assays, protein expression experiments and statistical analysis. XSC, CXH and LL coordinated and provided important suggestions including some reagents and critical reading the manuscript. All authors read and approved the final manuscript.

## Data Availability

The raw data supporting the conclusions of this manuscript will be made available by the authors to any qualified researcher.

## References

[1] Siegel RL , Miller KD , Jemal A . Cancer statistics, 2018. CA Cancer J Clin. 2018;68:7‐30. 29313949 10.3322/caac.21442

[2] Hirsch FR , Scagliotti GV , Mulshine JL , et al. Lung cancer: current therapies and new targeted treatments. Lancet. 2017;389:299‐311. 27574741 10.1016/S0140-6736(16)30958-8

[3] Cui CZ , Wen XS , Cui M , et al. Synthesis of solasodine glycoside derivatives and evaluation of their cytotoxic effects on human cancer cells. Drug Discov Ther. 2012;6:9‐17. 22460423

[4] Zhang X , Yan Z , Xu T , et al. Solamargine derived from *Solanum nigrum* induces apoptosis of human cholangiocarcinoma QBC939 cells. Oncol Lett. 2018;15:6329‐6335. 29731848 10.3892/ol.2018.8171 PMC5920861

[5] Burger T , Mokoka T , Fouche G , et al. Solamargine, a bioactive steroidal alkaloid isolated from *Solanum aculeastrum* induces non‐selective cytotoxicity and P‐glycoprotein inhibition. BMC Complement Altern Med. 2018;18:137. 29720141 10.1186/s12906-018-2208-7 PMC5930800

[6] Gu XY , Shen XF , Wang L , et al. Bioactive steroidal alkaloids from the fruits of *Solanum nigrum* . Phytochemistry. 2018;147:125‐131. 29306798 10.1016/j.phytochem.2017.12.020

[7] Tang Q , Zheng F , Wu J , et al. Combination of solamargine and metformin strengthens IGFBP1 gene expression through inactivation of Stat3 and reciprocal interaction between FOXO3a and SP1. Cell Physiol Biochem. 2017;43:2310‐2326. 29073599 10.1159/000484383

[8] Xiang S , Zhang Q , Tang Q , et al. Activation of AMPKalpha mediates additive effects of solamargine and metformin on suppressing MUC1 expression in castration‐resistant prostate cancer cells. Sci Rep. 2016;6:36721. 27830724 10.1038/srep36721 PMC5103223

[9] Fujisaka Y , Iwata T , Tamai K , et al. Long non‐coding RNA HOTAIR up‐regulates chemokine (C‐C motif) ligand 2 and promotes proliferation of macrophages and myeloid‐derived suppressor cells in hepatocellular carcinoma cell lines. Oncol Lett. 2018;15:509‐514. 29387231 10.3892/ol.2017.7322 PMC5768083

[10] Zheng P , Yin Z , Wu Y , et al. LncRNA HOTAIR promotes cell migration and invasion by regulating MKL1 via inhibition miR206 expression in HeLa cells. Cell Commun Signal. 2018;16:5. 29391067 10.1186/s12964-018-0216-3 PMC5796349

[11] Shang Z , Feng H , Cui L , et al. Propofol promotes apoptosis and suppresses the HOTAIR‐mediated mTOR/p70S6K signaling pathway in melanoma cells. Oncol Lett. 2018;15:630‐634. 29375720 10.3892/ol.2017.7297 PMC5766068

[12] Ren Y , Jia HH , Xu YQ , et al. Paracrine and epigenetic control of CAF‐induced metastasis: the role of HOTAIR stimulated by TGF‐ss1 secretion. Mol Cancer. 2018;17:5. 29325547 10.1186/s12943-018-0758-4 PMC5765658

[13] Loewen G , Jayawickramarajah J , Zhuo Y , et al. Functions of lncRNA HOTAIR in lung cancer. J Hematol Oncol. 2014;7:90. 25491133 10.1186/s13045-014-0090-4 PMC4266198

[14] Malek E , Jagannathan S , Driscoll JJ . Correlation of long non‐coding RNA expression with metastasis, drug resistance and clinical outcome in cancer. Oncotarget. 2014;5:8027‐8038. 25275300 10.18632/oncotarget.2469 PMC4226665

[15] Wu Y , Zhang L , Wang Y , et al. Long noncoding RNA HOTAIR involvement in cancer. Tumour Biol. 2014;35:9531‐9538. 25168368 10.1007/s13277-014-2523-7

[16] Bertoli G , Cava C , Castiglioni I . The potential of miRNAs for diagnosis, treatment and monitoring of breast cancer. Scand J Clin Lab Invest Suppl. 2016;245:S34‐S39. 27435502 10.1080/00365513.2016.1208444

[17] Li Y , Li Y , Chen Y , et al. MicroRNA‐214‐3p inhibits proliferation and cell cycle progression by targeting MELK in hepatocellular carcinoma and correlates cancer prognosis. Cancer Cell Int. 2017;17:102. 29151817 10.1186/s12935-017-0471-1 PMC5678695

[18] Zhu WS , Tang CM , Xiao Z , et al. Targeting EZH1 and EZH2 contributes to the suppression of fibrosis‐associated genes by miR‐214‐3p in cardiac myofibroblasts. Oncotarget. 2016;7:78331‐78342. 27823969 10.18632/oncotarget.13048 PMC5346642

[19] Li D , Liu J , Guo B , et al. Osteoclast‐derived exosomal miR‐214‐3p inhibits osteoblastic bone formation. Nat Commun. 2016;7:10872. 26947250 10.1038/ncomms10872 PMC4786676

[20] Ma L , Yang X , Wei R , et al. MicroRNA‐214 promotes hepatic stellate cell activation and liver fibrosis by suppressing Sufu expression. Cell Death Dis. 2018;9:718. 29915227 10.1038/s41419-018-0752-1 PMC6006298

[21] Giuliano CJ , Lin A , Smith JC , et al. MELK expression correlates with tumor mitotic activity but is not required for cancer growth. Elife. 2018;7:e32838. 29417930 10.7554/eLife.32838 PMC5805410

[22] Phatak P , Byrnes KA , Mansour D , et al. Overexpression of miR‐214‐3p in esophageal squamous cancer cells enhances sensitivity to cisplatin by targeting survivin directly and indirectly through CUG‐BP1. Oncogene. 2016;35:2087‐2097. 26234674 10.1038/onc.2015.271 PMC4740282

[23] Kuninty PR , Bojmar L , Tjomsland V , et al. MicroRNA‐199a and ‐214 as potential therapeutic targets in pancreatic stellate cells in pancreatic tumor. Oncotarget. 2016;7:16396‐16408. 26918939 10.18632/oncotarget.7651 PMC4941323

[24] Gagliardi PA , di Blasio L , Primo L . PDK1: a signaling hub for cell migration and tumor invasion. Biochim Biophys Acta. 2015;1856:178‐188. 26238471 10.1016/j.bbcan.2015.07.003

[25] Maegawa S , Chinen Y , Shimura Y , et al. Phosphoinositide‐dependent protein kinase 1 is a potential novel therapeutic target in mantle cell lymphoma. Exp Hematol. 2018;59:72‐81.e2. 29287939 10.1016/j.exphem.2017.12.006

[26] Ye XW , Yu H , Jin YK , et al. miR‐138 inhibits proliferation by targeting 3‐phosphoinositide‐dependent protein kinase‐1 in non‐small cell lung cancer cells. Clin Respir J. 2015;9:27‐33. 24405893 10.1111/crj.12100

[27] Feldman RI , Wu JM , Polokoff MA , et al. Novel small molecule inhibitors of 3‐phosphoinositide‐dependent kinase‐1. J Biol Chem. 2005;280:19867‐19874. 15772071 10.1074/jbc.M501367200

[28] Zheng N , Ding X , Sun A , et al. PDK1 activity regulates proliferation, invasion and growth of hemangiomas. Cell Physiol Biochem. 2015;36:1903‐1910. 26202351 10.1159/000430159

[29] Raimondi C , Falasca M . Targeting PDK1 in cancer. Curr Med Chem. 2011;18:2763‐2769. 21568903 10.2174/092986711796011238

[30] Shimobaba S , Taga S , Akizuki R , et al. Claudin‐18 inhibits cell proliferation and motility mediated by inhibition of phosphorylation of PDK1 and Akt in human lung adenocarcinoma A549 cells. Biochim Biophys Acta. 2016;1863:1170‐1178. 26919807 10.1016/j.bbamcr.2016.02.015

[31] Akizuki R , Shimobaba S , Matsunaga T , et al. Claudin‐5, ‐7, and ‐18 suppress proliferation mediated by inhibition of phosphorylation of Akt in human lung squamous cell carcinoma. Biochim Biophys Acta. 2017;1864:293‐302. 10.1016/j.bbamcr.2016.11.018 27884700

[32] Zheng F , Tang Q , Wu J , et al. p38alpha MAPK‐mediated induction and interaction of FOXO3a and p53 contribute to the inhibited‐growth and induced‐apoptosis of human lung adenocarcinoma cells by berberine. J Exp Clin Cancer Res. 2014;33:36. 24766860 10.1186/1756-9966-33-36 PMC4013801

[33] Zheng F , Wu J , Tang Q , et al. The enhancement of combination of berberine and metformin in inhibition of DNMT1 gene expression through interplay of SP1 and PDPK1. J Cell Mol Med. 2018;22:600‐612. 28840963 10.1111/jcmm.13347 PMC5742731

[34] Chen Y , Tang Q , Xiao Q , et al. Targeting EP4 downstream c‐Jun through ERK1/2‐mediated reduction of DNMT1 reveals novel mechanism of solamargine‐inhibited growth of lung cancer cells. J Cell Mol Med. 2017;21:222‐233. 27620163 10.1111/jcmm.12958 PMC5264151

[35] Yu S , Sheu HM , Lee CH *Solanum incanum* extract (SR‐T100) induces melanoma cell apoptosis and inhibits established lung metastasis. Oncotarget. 2017;8:103509‐103517. 29262580 10.18632/oncotarget.21508 PMC5732746

[36] Xiang S , Zou P , Tang Q , et al. HOTAIR‐mediated reciprocal regulation of EZH2 and DNMT1 contribute to polyphyllin I‐inhibited growth of castration‐resistant prostate cancer cells in vitro and in vivo. Biochim Biophys Acta. 2018;1862:589‐599. 10.1016/j.bbagen.2017.12.001 29221985

[37] Xiao Q , Zheng F , Wu J , et al. Activation of ERK and mutual regulation of Stat3 and SP1 contribute to inhibition of PDK1 expression by atractylenolide‐1 in human lung cancer cells. Cell Physiol Biochem. 2017;43:2353‐2366. 29073620 10.1159/000484387

[38] Kalalinia F , Karimi‐Sani I . Anticancer properties of solamargine: a systematic review. Phytother Res. 2017;31:858‐870. 28383149 10.1002/ptr.5809

[39] Hon KW , Abu N , Ab Mutalib NS , et al. miRNAs and lncRNAs as predictive biomarkers of response to FOLFOX therapy in colorectal cancer. Front Pharmacol. 2018;9:846. 30127741 10.3389/fphar.2018.00846 PMC6088237

[40] Shi J , Dong B , Cao J , et al. Long non‐coding RNA in glioma: signaling pathways. Oncotarget. 2017;8:27582‐27592. 28187439 10.18632/oncotarget.15175 PMC5432359

[41] Wu Y , Xiong Q , Li S , et al. Integrated proteomic and transcriptomic analysis reveals long noncoding RNA HOX transcript antisense intergenic RNA (HOTAIR) promotes hepatocellular carcinoma cell proliferation by regulating opioid growth factor receptor (OGFr). Mol Cell Proteomics. 2018;17:146‐159. 29079719 10.1074/mcp.RA117.000277 PMC5750844

[42] Cheng C , Qin Y , Zhi Q , et al. Knockdown of long non‐coding RNA HOTAIR inhibits cisplatin resistance of gastric cancer cells through inhibiting the PI3K/Akt and Wnt/beta‐catenin signaling pathways by up‐regulating miR‐34a. Int J Biol Macromol. 2018;107:2620‐2629. 29080815 10.1016/j.ijbiomac.2017.10.154

[43] Zhai N , Xia Y , Yin R , et al. A negative regulation loop of long noncoding RNA HOTAIR and p53 in non‐small‐cell lung cancer. Onco Targets Ther. 2016;9:5713‐5720. 27695348 10.2147/OTT.S110219 PMC5033503

[44] Zhang CG , Yin DD , Sun SY , et al. The use of lncRNA analysis for stratification management of prognostic risk in patients with NSCLC. Eur Rev Med Pharmacol Sci. 2017;21:115‐119. 28121347

[45] Wang R , Yan B , Li Z , et al. Long non‐coding RNA HOX transcript antisense RNA promotes expression of 14‐3‐3sigma in non‐small cell lung cancer. Exp Ther Med. 2017;14:4503‐4508. 29067125 10.3892/etm.2017.5041 PMC5647736

[46] Li N , Wang Y , Liu X , et al. Identification of circulating long noncoding RNA HOTAIR as a novel biomarker for diagnosis and monitoring of non‐small cell lung cancer. Technol Cancer Res Treat. 2017;16:1060‐1066. 10.1177/1533034617723754 PMC5762071 28784052

[47] Li CH , Xiao Z , Tong JH , et al. EZH2 coupled with HOTAIR to silence MicroRNA‐34a by the induction of heterochromatin formation in human pancreatic ductal adenocarcinoma. Int J Cancer. 2017;140:120‐129. 27594424 10.1002/ijc.30414

[48] Lai Y , He S , Ma L , et al. HOTAIR functions as a competing endogenous RNA to regulate PTEN expression by inhibiting miR‐19 in cardiac hypertrophy. Mol Cell Biochem. 2017;432:179‐187. 28316060 10.1007/s11010-017-3008-y

[49] Wang Z , Wei M , Zhang Q , et al. Comparison of high‐flexion and conventional implants in total knee arthroplasty: a meta‐analysis. Med Sci Monit. 2015;21:1679‐1686. 26057659 10.12659/MSM.893112 PMC4467602

[50] Liu XH , Sun M , Nie FQ , et al. Lnc RNA HOTAIR functions as a competing endogenous RNA to regulate HER2 expression by sponging miR‐331‐3p in gastric cancer. Mol Cancer. 2014;13:92. 24775712 10.1186/1476-4598-13-92 PMC4021402

[51] Bartel DP . MicroRNAs: genomics, biogenesis, mechanism, and function. Cell. 2004;116:281‐297. 14744438 10.1016/s0092-8674(04)00045-5

[52] Ambros V . The functions of animal microRNAs. Nature. 2004;431:350‐355. 15372042 10.1038/nature02871

